# Fault Scarp Dating Tool - a MATLAB code for fault scarp dating using in-situ chlorine-36 supplemented with datasets of Yavansu and Kalafat faults

**DOI:** 10.1016/j.dib.2019.104476

**Published:** 2019-09-16

**Authors:** Dmitry Tikhomirov, Nasim Mozafari Amiri, Susan Ivy-Ochs, Vasily Alfimov, Christof Vockenhuber, Naki Akçar

**Affiliations:** a Institute of Geological Sciences, University of Bern, Baltzerstrasse 1+3, 3012, Bern, Switzerland; b Laboratory of Ion Beam Physics, ETH Zurich, Otto-Stern-Weg 5, 8093, Zurich, Switzerland; c Department of Geography, University of Zurich, Winterthurerstrasse 190, 8057, Zurich, Switzerland

**Keywords:** Normal fault scarp, Exposure dating, Cosmogenic chlorine-36, Matlab, Western Turkey

## Abstract

We publish a MATLAB code used to analyze concentration profile of cosmogenic ^36^Cl accumulated in-situ through a rupture history of the fault scarps in western Turkey (Mozafari et al., 2019). The code is a version of the forward modeling Matlab code -Fault Scarp Dating Tool- (Tikhomirov, 2014). The code models a ^36^Cl profile accumulated in the fault scarp surface through a guessed rupture history, and compares the modeled and measured ^36^Cl profiles with statistical tests. Rupture histories are randomly generated in bounded solution space using Monte-Carlo method or optimized using Random Walk algorithm to achieve the best fit of the modeled and measured ^36^Cl profiles. The code has a user-friendly interface, a build-in help and an example of input data.

**Table:** Specifications Table

Subject	*Geology*
Specific subject area	*Geochronology, Tectonics, Cosmogenic Nuclide Dating*
Type of data	*Modeling and data analysis program (Matlab)* *Input data (MS Excell)* *Database files (Matlab)*
How data were acquired	*The code is written in MATLAB numerical computing environment* *The datasets for analysis were acquired with dimensional measurement in the field, ICP-MS and AMS*
*Data format*	*MATLAB computing code* *Numerical dataset*
Parameters for data collection	*Well-preserved fault surface with less amount of weathering and erosion were chosen for continuous sampling from the ground to top of the fault surface.*
Description of data collection	15 cm *wide by* 10 cm *high samples were collected with a chisel and hammer from the* 3 cm *outermost surface of the fault parallel to the slip direction.*
Data source location	*Kuşadası, western Turkey*
Data accessibility	*The complete data is presented with this article only, but partly in*[1]
Related research article	*N. Mozafari, Ö. Sümer, D. Tikhomirov, S. Ivy-Ochs; V. Alfimov, C. Vockenhuber, U. İnci, H. Sözbilir; N. Akçar, Holocene seismic activity of the Priene-Sazlı Fault revealed by cosmogenic*^*36*^*Cl, western Anatolia, Turkey, Turkish Journal of Earth Sciences, 28 (2019) 410–437.*https://doi.org/10.3906/yer-1810-6

**Table:** 

**Value of the data** • The presented MATLAB computing code can be useful for analysis of cosmogenic chlorine-36 accumulated by a normal limestone fault scarp in order to reconstruct the rupture history of the fault. • The code could be interested to geoscientists applying the dating with cosmogenic nuclides to landforms with complex and dynamical geometry. • Modular structure of the code allows adjusting the code to other cosmogenic nuclides, e.g. 10Be, 14C, 26Al, as well as to different landform geometries. • The full input data of the Kalafat and Yavansu fault scarps can be used to cross-check or reanalyze the results of the main publication [1].

## 1 Data

The following data are presented:

1.1 ‘FSDT code’ folder contains the Fault Scarp Dating Tool code files:
  1.1.1 _how-to_run_the_code.txt – the text file, which explains how-to run the code.
  1.1.2 _List of code's functions and files – the.pdf file with a list of all files of the code.
  1.1.3 angdist.m – see CRONUS-Earth Al-26/Be-10 exposure age calculator, MATLAB function reference, Version 2: November 2007. http://hess.ess.washington.edu/math/docs/al_be_v2/al_be_fctn_desc/
  1.1.4 antatm.m – see CRONUS-Earth Al-26/Be-10 exposure age calculator, MATLAB function reference, Version 2: November 2007. http://hess.ess.washington.edu/math/docs/al_be_v2/al_be_fctn_desc/
  1.1.5 BalcoScaling.m – the compilation of functions make_al_be_consts_v22.m and get_al_be_age.m. See CRONUS-Earth Al-26/Be-10 exposure age calculator, MATLAB function reference, Version 2: November 2007. http://hess.ess.washington.edu/math/docs/al_be_v2/al_be_fctn_desc/
  1.1.6 beta_ebar.m – a modification of function P_mu_total.m. See CRONUS-Earth Al-26/Be-10 exposure age calculator, MATLAB function reference, Version 2: November 2007. http://hess.ess.washington.edu/math/docs/al_be_v2/al_be_fctn_desc/
  1.1.7 Calcnbins.m – Calculate the "ideal" number of bins to use in a histogram, using a choice of methods.
  1.1.8 cleanXlsSheets.m – cleans an XLS-file content. http://www.mathworks.com/matlabcentral/answers/642-how-do-i-clear-the-contents-of-excel-by-sheet/
  1.1.9 colluvium – a sub function to calculate shielding under colluvium.
  1.1.10 cont_mode.m – finds the modal value of a continuously distributed variable.
  1.1.11 d2r.m – see CRONUS-Earth Al-26/Be-10 exposure age calculator, MATLAB function reference, Version 2: November 2007. http://hess.ess.washington.edu/math/docs/al_be_v2/al_be_fctn_desc/
  1.1.12 Database_Maker_Help.txt - the help file of Database Maker.
  1.1.13 Database_Maker_Help.txt – the help file of Database Maker.
  1.1.14 DBmaker.m – creates a database for reconstruction of the exposure history of the fault scarp.
  1.1.15 desilets2006sp.m – see CRONUS-Earth Al-26/Be-10 exposure age calculator, MATLAB function reference, Version 2: November 2007. http://hess.ess.washington.edu/math/docs/al_be_v2/al_be_fctn_desc/
  1.1.16 dunai2001sp.m – see CRONUS-Earth Al-26/Be-10 exposure age calculator, MATLAB function reference, Version 2: November 2007. http://hess.ess.washington.edu/math/docs/al_be_v2/al_be_fctn_desc/
  1.1.17 fit1decay.m – approximates data with one exponential decay function.
  1.1.18 FSDT_Database_Maker.fig – the layout description of Database Maker interface.
  1.1.19 FSDT_Database_Maker.m – the control code of Database Maker interface.
  1.1.20 FSDT_Simulator.fig – the layout description of Simulator interface.
  1.1.21 FSDT_Simulator.m – the control code of Simulator interface.
  1.1.22 fun2decay.m – returns the value of sum of two exponential decay functions.
  1.1.23 fun3decay.m – returns value of sum of three exponential decay functions.
  1.1.24 get_rand.m – generates a vector of random values following uniform or normal distribution.
  1.1.25 Histogram_Overview.m – the layout description and the control code of Histogram Overview window.
  1.1.26 lifton2006sp.m – see CRONUS-Earth Al-26/Be-10 exposure age calculator, MATLAB function reference, Version 2: November 2007. http://hess.ess.washington.edu/math/docs/al_be_v2/al_be_fctn_desc/
  1.1.27 Main_Result.m – the layout description and the control code of Main Result window.
  1.1.28 MonteCarloScarp.m – generates random rupture histories and collects the histories with fit chi-squared value below the threshold.
  1.1.29 muon_flux.m – a modification of function P_mu_total.m. See CRONUS-Earth Al-26/Be-10 exposure age calculator, MATLAB function reference, Version 2: November 2007. http://hess.ess.washington.edu/math/docs/al_be_v2/al_be_fctn_desc/
  1.1.30 NCEP2.mat – see CRONUS-Earth Al-26/Be-10 exposure age calculator, MATLAB function reference, Version 2: November 2007. http://hess.ess.washington.edu/math/docs/al_be_v2/al_be_fctn_desc/
  1.1.31 NCEPatm_2.m – see CRONUS-Earth Al-26/Be-10 exposure age calculator, MATLAB function reference, Version 2: November 2007. http://hess.ess.washington.edu/math/docs/al_be_v2/al_be_fctn_desc/
  1.1.32 nucl_data.mat – the data file with nuclear physical constants.
  1.1.33 Parameter_Window.m – the layout description and the control code of Resulting Parameter window.
  1.1.34 PMag_Mar07.mat – see CRONUS-Earth Al-26/Be-10 exposure age calculator, MATLAB function reference, Version 2: November 2007. http://hess.ess.washington.edu/math/docs/al_be_v2/al_be_fctn_desc/
  1.1.35 PointShield_mu.m – calculates the scaled flux of fast muons at the point of footwall.
  1.1.36 PointShield_sp.m – calculates the shielding factor for high-energy neutrons at the point of footwall.
  1.1.37 PointShield_sp_coll.m – calculates the shielding factor for high-energy neutrons at the point of colluvium surface.
  1.1.38 PointShield_sp_th.m – calculates the apparent attenuation length in the footwall surface below colluvium.
  1.1.39 Probability_Density_Plot.m – the layout description and the control code of Probability Density Plot window.
  1.1.40 r2d.m – see CRONUS-Earth Al-26/Be-10 exposure age calculator, MATLAB function reference, Version 2: November 2007. http://hess.ess.washington.edu/math/docs/al_be_v2/al_be_fctn_desc/
  1.1.41 RandomWalkScarp.m – optimizes the rupture history to achieve a minimum of the fit chi-squared value.
  1.1.42 round_from_zero.m – rounds a number to the next higher number.
  1.1.43 Scarp_Visual_Check.m – the layout description and the control code of Visual Check window of the scarp cross-section.
  1.1.44 ScarpSimulator.m – calculates accumulated 36Cl profile according to the rupture history.
  1.1.45 sec2hhmmss.m – converts seconds into HH.MM.SS format.
  1.1.46 Simulator_Help.txt – the help file of Simulator.
  1.1.47 sky – a sub function of one formula.
  1.1.48 spall – a sub function of one formula.
  1.1.49 start – the function to start FSDT with the command ‘start’.
  1.1.50 stone2000.m – see CRONUS-Earth Al-26/Be-10 exposure age calculator, MATLAB function reference, Version 2: November 2007. http://hess.ess.washington.edu/math/docs/al_be_v2/al_be_fctn_desc/
  1.1.51 stone2000Rcsp.m – see CRONUS-Earth Al-26/Be-10 exposure age calculator, MATLAB function reference, Version 2: November 2007. http://hess.ess.washington.edu/math/docs/al_be_v2/al_be_fctn_desc/
  1.1.52 ThermEpithermNeutronCoef.m – calculates the attenuation lengths and coefficients of two group diffusion approximation of thermal and epithermal neutrons.
  1.1.53Topo_Visual_Check.m – the layout description and the control code of Visual Check window of the topographic shielding.
  1.1.54 TpToTopo.m – converts the bar approximation or the broken-line approximation of the horizon line into the binary matrix of the hemisphere mesh.
  1.1.55 Ugol.m – a sub function of one formula.
  1.1.56 About.txt – about text file.
1.2 ‘KAL-Data’ folder contains the dataset of the Kalafat fault scarp including the following files:
  1.2.1 KAL-Input.xlsx – the input sheet of the chemical composition and geometry of Kalafat sample set.
  1.2.2 Database KAL.mat – the database of Kalafat dataset.
  1.2.3 Database KAL_input_data.txt – the input data of FSDT_Database_Maker used to produce the database ‘Database KAL.mat’ of Kalafat dataset.
  1.2.4 Screenshot of FSDT DB interface KAL.jpg – the screenshot of FSDT_Database_Maker interface with entered input data of Kalafat dataset.
1.3 ‘YAV-Data’ folder contains the dataset of the Yavansu fault scarp including the following files:
  1.3.1 YAV -Input.xlsx – the input sheet of the chemical composition and geometry of Yavansu sample set.
  1.3.2 Database YAV.mat – the database of Yavansu dataset.
  1.3.3 Database YAV _input_data.txt – the input data of FSDT_Database_Maker used to produce the database ‘Database YAV.mat’ of Yavansu dataset.
  1.3.4 Screenshot of FSDT DB interface YAV.jpg – the screenshot of FSDT_Database_Maker interface with entered input data of Yavansu dataset.

## 2 Experimental design, materials, and methods

The full description of the methods used to acquire the datasets of the Kalafat and Yavansu fault scarps is published in the main publication [1]. The description includes the sampling in the field, mechanical and chemical preparation and details on sample measurement with accelerator mass-spectrometry. In this publication we would like to present the computation code used to analyze the datasets.

Fault Scarp Dating Tool (FSDT) is the MATLAB® code for fault scarp dating and earthquake history reconstruction. The code applies forward modeling and Monte-Carlo algorithms to determine the most probable displacement history: the age of displacement events, the displacement values (slips) and the uncertainty of ages and slips. Displacement histories are randomly generated, transformed to ^36^Cl concentration profile and compared to measured concentrations with goodness-of-fit tests. The current code is an improved and updated version of the previous FSDT code published in [2].

The program has build-in help files and a user-friendly graphic interface. Output of the program is presented graphically and numerically and can be saved in a variety of file formats. The program was tested on MATLAB® versions 8.5 and higher. To run the code, a MATLAB® assembly should include Parallel Computing Toolbox™. The source code can be distributed and modified under Creative Commons Attribution-NonCommercial-ShareAlike 3.0 Unported License.

### 2.1 Production of cosmogenic ^36^Cl in the fault scarp

A mathematical model of cosmogenic isotope production in a fault scarp is essential to transform exposure history into ^36^Cl distribution. Classic surface exposure dating approach for calculation of accumulated isotope concentration cannot be applied to a fault scarp, due to complex and changing geometry of the scarps. In our previous publication, we formulated a mathematical model for shielding factor calculation in the fault scarp geometry and gave example with high-energy neutrons [3]. In our new code, this model is broadened to all particles which share ^36^Cl production: high-energy neutrons, thermal and epithermal neutrons, fast and negative muons. In this new model, high-energy neutrons flux is calculated in the form of the shielding factors over the sample stripe volume. Relying on high-energy neutron attenuation length perpendicular to the scarp surface, thermal and epithermal neutron fluxes are calculated over the samples above colluvium using two-group diffusion approach [4]. To derive these fluxes for samples under colluvium, high-energy neutron flux over the colluvium surface and attenuation length of high-energy neutrons perpendicular to colluvium surface are additionally precalculated. Fast muon flux is calculated over the sample stripe with a code modified after [5] to fit fault scarp geometry. This modified code follows the muon production model by [6], [7] and the muon scaling by [5]. The rate of stopped negative muons over the sample stripe is calculated as a derivative of one exponential approximation of fast muon flux with respect to scarp geometry. Using fast and negative muon fluxes, the production of thermal neutrons by muons is calculated. Production of thermal neutrons from decay of ^238^U and (α,n) reactions by U and Th series is calculated following [8]. In total, ^36^Cl production rate on Ca, K, Ti, Fe and ^35^Cl by all significant reaction channels are derived over the sample stripe volume. These calculations of the production rate are done for the different relative positions of footwall and colluvial wedge, which correspond to periods of quiescence between ruptures.

### 2.2 Two-step computation

Direct modeling of one exposure history of a fault scarp takes about a half of an hour with our code on a modern office PC. The computation is done at a huge amount of points of the scarp: several points are required to average concentration over the sample volume, the sample stripe usually contains dozens of samples, and few fault scarp configurations interchange each other through scarp history. To reduce computation time and to apply the Monte-Carlo method, we divided the computation process into two stages.

At the first stage, the particle fluxes and the shielding factors are calculated at nodes of 3-dimentional grid, which covers all possible positions of the sample stripe and all possible fault scarp configurations in the past. The dimensions of the grid are the depth perpendicular to the scarp surface, the position along the fault scarp height and the fault scarp height along its surface. Additionally, sample coefficients for all production channels and scaling factors for high-energy neutrons [5] are calculated at this stage. The result of the first stage is saved in a separate file and serves as a database for the second calculation stage.

At the second stage, the code randomly generates an exposure history with a predefined number of displacement events. The particle fluxes and the shielding factors are interpolated to each particular scarp configuration using the database. ^36^Cl distributions accumulated during each scarp configuration are calculated and merged to represent the exposure history. In comparison to direct modeling, the modeling of an exposure history with the database support takes about a tenth of a second. Creation of one database requires several hours, but in the light of numerous simulations with the Monte-Carlo method, two-stage computation gives tremendous time profit.

### 2.3 Monte-Carlo and Random Walk algorithms

FSDT code applies simple Monte-Carlo or Random Walk algorithms to search the most probable exposure history. The ages of displacement events, the colluvium positions, the beginning of exposure and the erosion rate are the desired parameters which uniquely describe the exposure history in our model. The ages of displacement events are equal to ages of rupture events for normal fault scarp which configuration was affected by ruptures only. Each rupture event is represented by one earthquake or by multiple earthquakes occurred in the short time span which cannot be resolved by the method. Due to aspects of the modeling, the position of colluvium level prior to displacement event is more convenient to operate than the normal component of slip. The same reference point and the position axis which were defined for sample position on the scarp are used for the position of colluvium level. The colluvium position prior to the event can be also calculated as the total cumulative slip less the cumulative slip prior to the event. The beginning of exposure is an age when the production of ^36^Cl in the sample stripe becomes appreciable by the dating method. This modeling parameter cannot be certainly referred to any dating or geological characteristic of the fault scarp; the closest characteristic would be the exposure age of the fault scarp top.

The Random Walk algorithm of the code generates new exposure history by variation of each desired parameter according to a normal distribution. Parameter values of previous exposure history are used as means of normal distributions, while deviations are fixed through the run of the algorithm. The reduced chi-square value is calculated for each generated exposure history to indicate the goodness of fit of the modeled and measured ^36^Cl profile:

(1) $$\chi^{2}=\frac{1}{n-k}\sum_{i=1}^{n}\left[\frac{{\left(Ne_{i}-Nm_{i}\right)}^{2}}{Ne_{i}^{2}\cdot \left(\sigma e_{i}^{2}+\sigma p_{i}^{2}\right)}\right]$$

where *Ne*_*i*_ is the measured ^36^Cl concentration, atoms g^−1^, *Nm*_*i*_ is the modeled ^36^Cl concentration, atoms g^−1^, *σe*_*i*_ is the relative standard error of AMS measurement of ^36^Cl concentration, *σp*_*i*_ is the relative standard error of parent element concentrations, *n* is the number of samples, and *k* is the number of calculated parameters.

Our code offers two types of conditions which can be set on chi-square value in the Random Walk mode: threshold of chi-square value and minimization of chi-square value. At the threshold condition, new exposure history is saved and accepted as the next point of algorithm if its chi-square value is less than or equal to the threshold. At the minimization condition, all generated exposure histories are saved, while new exposure history is accepted as the next point if its chi-square value satisfies following condition:

(2) $$\chi_{new}^{2}<\frac{\chi_{\min}^{2}}{U^{\frac{1}{\alpha }}}$$

where $\chi_{\min}^{2}$is the minimal found chi-square value;$\chi_{new}^{2}$ is the chi-square value of new exposure history; *U* is uniformly distributed value on the open interval (0,1); and *α* is the decision parameter.

The Monte-Carlo algorithm of the code randomly samples solution space bounded by uniform and/or normal distributions of desired parameters. Uniform distribution is preferred when no independent information on a parameter value is available, while normal distribution is used to fix a parameter relying on independent data, for instance the age of the historical earthquake or weathering boundary. Only chi-square threshold condition is available in Monte-Carlo mode. Exposure histories with chi-square value less than or equal to the given threshold are saved, while the rest are rejected. The Monte-Carlo algorithm of the code is less advanced than the Random Walk algorithm and requires longer time to find the best-fit solution.

Both Monte-Carlo or Random Walk algorithms cannot perform direct optimization of the number of events. Therefore, scenarios with different number of events should be considered to find the most probable exposure history. However, indirect optimization of the number of events is an inherent property of the code; extra events tend to converge through the simulation. The chi-square indicator could be insufficient to distinguish between scenarios with different number of events. To support it, the corrected Akaike information criterion (AICc) is calculated as follows,

(3) $$AICc=\left(n-k\right)\cdot \chi^{2}+\frac{2kn}{n-k-1}$$

Such formulation of AICc differs from the standard definition of AICc by a constant, which can be omitted within analysis of one data set. As it seen from the formula above, AICc is more strict indicator to additional modeling parameters than chi-square. In our model the minimal value of AICc indicates the exposure history with the optimal number of events.
